# The Texas State Medical Meeting

**Published:** 1919-05

**Authors:** 


### The Texas State Medical Meeting.

The Texas State Medical Meeting at Waco, this month, was one of the best yet held in the history of the association. The meeting of the State Medical Association was preceded by a joint session of The Texas Railway Surgeon's Association and the Texas Roentgen Ray Society, the meeting being occupied with the reading and discussion of technical papers.

Officers elected by the Texas Roentgen Ray Society are: President, Dr. Bacon Saunders, Fort Worth; vice president, Dr. C. S. Venable, San Antonio, and secretary and treasurer, Dr. G. H. Reed, Beaumont.

Officers elected by the Texas Roentgen Ray Society are: President, Dr. J. W. Torbett, Marlin; vice president, Dr. J. T. Vanzendt, Houston; secretary and treasurer, Dr. S. D. Whitten, Greenville. The feature of the joint meeting was an illustrated lecture by Dr. J. T. Case of Battle Creek, who served with the American Expeditionary Forces in France.

The opening session of the convention consisted of the address of welcome by Mayor McCullough, followed by an invitation by Rev. R. E. Goodrich, of the Austin Street Methodist church. Then, Dr. W. O. Wilkes, gave an address of welcome on behalf of the physicians of the city, and Dr. S. P. Rice, of Marlin, the president, delivered his annual address.

The programme, as originally published, was strictly followed, except in a few cases where the parties assigned to read papers could not be present. It may be stated that most of the papers evidenced much serious study of the subjects and will result in good to the profession.

Houston was selected for the 1920 meeting place. Dr. T. T. Jackson, of San Antonio, was elected president-elect. Vice presidents elected are Doctors E. T. Aber, Dallas; George H. Lee, Galveston, and W. L. Crosthwait, Waco.

Dr. Holman Taylor, Fort Worth,, was re-elected secretary, and Dr. Wilmer L. Allison, Fort Worth, treasurer.

Councilors of medical defense: Doctors W. A. King, San Antonio, to succeed himself; W. D. Jones, Dallas, to succeed H. W. Cummings, resigned.

Dr. C. F. Cantrell, of Greenville, was elected trustee to fill out the unexpired term of Dr. T. T. Jackson, who was made president-elect. Dr. C. M. Alexander of Coleman was elected trustee.

The following doctors were elected as district councillors:

R. S. Killough, Amarillo, third; C. S. Venable, Amarillo, fifth; R. U. Painter, Corpus Christi, sixth; W. C. McElhannon, Belton, twelfth; C. E. Seale, Dangerfield, fifteenth; P. C. Coleman, Colorado, second and Joe E. Dildy, Lampasas, fourth.

### Delegates to A. M. A.

Doctors W. W. Ralston, Houston; W. B. Russ, San Antonio, and C. E. Cantrell, were elected delegates to the American Medical Association, with Doctors S. P. Rice, Marlin; C. E. Durham, Hico, and W. P. White, Henderson, as alternates, respectively.

Dr. I. C. Chase was elected to membership on the council on legislation and public instruction.

### Adopt Important Resolutions.

Among the resolutions passed by the delegation are the following: That the civil authorities of the state continue the health measures that were enacted by military authorities and asked legislature to pass proper laws to that effect; favoring the erection and maintenance by the state of an appropriate sanitorium to provide care and treatment for negroes suffering with tuberculosis as soon as possible so that those now uncared for patients may not spread the white plague among the citizenship of the state generally.

### Trial of Insane by Doctors.

Another resolution requests Gov. W. P. Hobby to submit an amendment providing for the trial of the insane by a jury of physicians instead of laymen, to be voted at the next general election, "because the trial of lunatics as now conducted in Texas is not only subversive of their rights as helpless human beings but is obsolete and unworthy of the enlightened and progressive age."

A rising vote of thanks was given to the physicians of the McLennan County Medical society and its auxiliary, the press, the Chamber of Commerce, the Y. M. C. A., the Knights of Columbus, the City Commission, the house of delegates, the hotels and to the people of Waco, for their welcome and entertainment. Other resolutions were: Soliciting the legislature to make sufficient appropriations to permit the essential work of the state board of health through its engineering and rural sanitation bureau; asking the board of directors to appropriate and set aside an amount sufficient to pay the state association dues of all members in arrears who are now or were in the service Jan. 1, 1919; recommending that the various county societies change their organic law so that the county secretaries shall be elected for a term of five years; recommending that the directors have a history written of Texas medicine and Texas physicians.

### Memorial to Members.

"The fittest place where man can die is where he dies for man," proclaimed Dr. Marvin L. Graves, of Galveston, in his tribute Wednesday afternoon in memory of the members of the State Medical association of Texas who had died since the last annual convention.

Dr. Graves paid a heart-gripping tribute to the memory of his fallen comrades, many of whom he mentioned by name.

President-elect Dr. T. T. Jackson, of San Antonio, in his address on "The Part of Texas Doctors in the War" informed the association that there were commissioned in the army from Texas 825 physicians; in the navy, 50, and in the national guards 52, making a total of 927 men from Texas out of a national total of 32,000 medical officers.

---

Dr C. S. Brown, when relieved from the service, is arranging to resume his practice at Garland, Texas.

Dr. C. M. Kent has been discharged from the service where he was a captain, and will locate in Kenedy again.

Dr. M. O. Rea, after spending eight months in Italy on duty in Roentgen Ray service, has resumed his practice in Dallas.

Dr. John G. McLaurin, who was stationed nine months in a hospital at Bordeaux, France, is back at home in Dallas.

Dr. H. L. Warwick, who was chief of the ear, nose and throat work at a war hospital in Baltimore, will resume his practice in Fort Worth.

Dr. J. V. Wright, thirty-three years old, superintendent of Woodlawn Hospital, Dallas, died recently as a result of an operation.

Dr. H. F. Connally, of Marlin, stationed at Chaumont, France, has been promoted from Major to Lieutenant-Colonel.

